# Temperature‐Triggered Adhesive Bioelectric Electrodes with Long‐Term Dynamic Stability and Reusability

**DOI:** 10.1002/advs.202300793

**Published:** 2023-05-18

**Authors:** Huiting Lai, Yan Liu, Yin Cheng, Liangjing Shi, Ranran Wang, Jing Sun

**Affiliations:** ^1^ State Key Laboratory of High Performance Ceramics and Superfine Microstructure Shanghai Institute of Ceramics Chinese Academy of Sciences 1295 Ding Xi Road Shanghai 200050 China; ^2^ Center of Materials Science and Optoelectronics Engineering University of Chinese Academy of Sciences 19 Yuquan Road Beijing 100049 China; ^3^ School of Chemistry and Materials Science Hangzhou Institute for Advanced Study University of Chinese Academy of Sciences 1 Sub‐lane Xiangshan Hangzhou 310024 China

**Keywords:** bioelectric electrodes, dynamic monitoring, long‐term monitoring, reusability, temperature‐triggered adhesion switch

## Abstract

Bioelectric electrodes with low modulus and high adhesion have been intensively pursued, as they afford conformal and strong bonding at skin‐electrode interface to improve the fidelity and stability of electrophysiological signals. However, during detachment, tough adhesion can cause pain or skin allergy; worse still, the soft electrodes can suffer damage due to excessive stretch/torsion, hampering long‐term, dynamic, and multiple uses. Herein, a bioelectric electrode is proposed by transferring silver nanowires (AgNWs) network to the surface of bistable adhesive polymer (BAP). The phase transition temperature of BAP is tuned to be slightly below skin temperature at 30 °C. Triggered by skin heat, the BAP electrode achieves low modulus and high adhesion within seconds, allowing robust skin‐electrode interface under dry, wet, and body‐moving conditions. Ice bag treatment can dramatically stiffen the electrode and reduce the adhesion, which allows painless detachment and avoids electrode damage. Meanwhile, the AgNWs network with biaxial wrinkled microstructure remarkably promotes the electro‐mechanical stability of the BAP electrode. The BAP electrode successfully combines long‐term (7 days) and dynamic (body movements, sweat, underwater) stability, reusability (at least ten times), and minimized skin irritation during electrophysiological monitoring. The high signal‐to‐noise ratio and dynamic stability are demonstrated in the application of piano‐playing training.

## Graphical abstract

A bioelectric electrode is developed by incorporating a temperature‐sensitive phase‐transition polymer and a biaxially pre‐strained AgNWs percolation network. The bioelectric electrode exhibits a quick and dramatic stiffness/adhesion switch around skin temperature, which enables stable electrophysiological monitoring in long‐term and dynamic states, and allows painless and non‐destructive detachment and reusability.

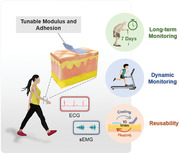

## 1 Introduction

With the development of wearable devices and wireless communication, real‐time monitoring of bioelectric signals, such as electrocardiogram (ECG), electromyography (EMG), and electroencephalogram (EEG) has been realized to help better understand the pathological and physiological conditions of the human body.^[^ ^1^, ^2^, ^3^, ^4^ ^]^ In addition, recognition of EMG or EEG signals can assist disabled people to type^[^ ^5^ ^]^ or grasp objects,^[^ ^6^ ^]^ and also provide a wide range of applications in human‐computer interaction.^[^ ^7^, ^8^ ^]^ These applications require bioelectric electrodes to be able to accurately identify complex and weak bioelectric signals, sustain reusable and long‐duration monitoring, and remain stable against dynamic activities and sweat disturbances.

Bioelectric electrodes can generally be divided into wet, semi‐dry, and dry electrodes.^[^ ^9^ ^]^ Gel electrodes are typical wet electrodes. Commercial Ag/AgCl gel electrodes can be used to collect high‐quality bioelectric signals under static and short‐time events. However, the easy drying of the electrolyte gel layer reduces its electrical conductivity and interfacial adhesion with the skin during long‐term use, resulting in signal distortion.^[^ ^10^ ^]^ The water retention of the gel electrode can be improved by introducing hygroscopic materials such as glycerol,^[^ ^11^ ^]^ phytic acid,^[^ ^12^ ^]^ and LiCl salts^[^ ^13^ ^]^ to extend its stable operation life up to 72 h at most. However, gel electrodes that can work reliably for more than 3 days are rarely reported. In addition, due to the presence of hydrophilic groups in these gels, they tend to swell and lose the robust bonding with skin when exposed to water, which limits their practical use for underwater and sweating events.^[^ ^14^, ^15^ ^]^ At the same time, issues such as allergic reactions caused by long‐term adhesion also need to be addressed.^[^ ^16^ ^]^

Semi‐dry electrodes can store and release electrolyte solution (such as NaCl solution) from reservoirs, which solve the problem of water loss in long‐term monitoring.^[^ ^17^, ^18^, ^19^ ^]^ The semi‐dry EEG electrodes with sponge structure as reservoirs have low skin‐electrode impedance and high accuracy of EEG signals, which is comparable to commercial gel electrodes.^[^ ^20^ ^]^ This stems from the high flexibility of the sponge structure that allows the electrode to bypass the hair to form a conformal contact with the scalp. However, the reservoir of semi‐dry electrodes is usually bulky and the electrolyte needs to be manually loaded, which limits the convenient daily use.

Gel‐free dry electrodes can overcome the problem of signal distortion caused by gel dehydration. However, the mechanical mismatch between the soft skin and relatively rigid dry electrode usually results in high contact impedance. Worse still, their signals are highly susceptible to motion artifacts. An effective strategy to address these issues is to promote the flexibility of the dry electrodes. For example, fabric electrodes can be highly flexible, which are conducive to long‐term monitoring.^[^ ^21^, ^22^ ^]^ Unfortunately, their contact impedance is higher than that of commercial gel electrodes due to poor conformity with skin and the small contact area of porous fabric structures. The flexibility of dry electrodes can also be improved by reducing the thickness or Young's modulus.^[^ ^23^ ^]^ Zhao et al.^[^ ^24^ ^]^ prepared an ultra‐thin dry electrode with a thickness of about 100 nm, and the enhanced conformability to skin contributed to higher signal‐to‐noise ratio (SNR) of 23 ± 0.7 dB in comparison with 19 ± 0.5 dB of the Ag/AgCl electrode. Tang et al.^[^ ^25^ ^]^ introduced glycerol and polysorbate into PEDOT: PSS‐based electrode to reduce the Young's modulus to 80 kPa (similar to that of skin), giving rise to bioelectric signal with high SNR (≈35 dB).

Although the promoted flexibility leads to high quality signal through optimized interface conformability and contact area,^[^ ^26^ ^]^ the relative movement at the skin‐electrode interface still induces interference signals, representing a most intractable problem especially during long‐term and dynamic monitoring. Therefore, it is critical to maintain a robust interface bonding for high‐fidelity bioelectric signals.^[^ ^27^ ^]^ One way is to chemically modify the electrode material to form attractive interaction at the interface, such as hydrogen bond, dynamic covalent bond, electrostatic interaction, etc.^[^ ^28^, ^29^ ^]^ Another approach is to raise the adhesive force by means of biomimetic micropillars or sucker structures.^[^ ^30^, ^31^, ^32^ ^]^ However, such boosted extra‐strong adhesion inflicts irritation, pain, or even allergy to the skin during detachment.^[^ ^33^, ^34^ ^]^ Besides, electrodes with low modulus and high adhesion are particularly prone to excessive deformation when detached, which leads to irreversible damage to the electrode, making it impossible for continuous high‐quality signal collection and electrode reusability. To the best of our knowledge, bioelectric electrodes that successfully combine long‐term (>3 days) and dynamic (body movements, sweat, underwater, et al.) stability, reliable reusability, and minimized skin irritation have rarely been reported.

Here, we proposed a dry electrode with on‐demand interfacial adhesion switch based on bistable adhesive polymer (BAP)^[^ ^35^ ^]^ and highly conductive silver nanowires (AgNWs) network. The BAP electrodes can rapidly switch between two states by changing the temperature. When triggered by skin heat, the BAP electrodes achieve high flexibility and strong adhesion, and adhere conformally and firmly to the skin even in wet conditions. Through cold water or ice bag treatment, the electrodes can be easily detached from the skin due to significantly increased modulus and reduced adhesion. Compared with other triggering methods (pH,^[^ ^36^ ^]^ humidity,^[^ ^33^ ^]^ magnetism,^[^ ^37^ ^]^ etc.), temperature control is more convenient and easier. A wrinkled microstructure is engineered at the BAP electrode surface to extend the stretchability of the AgNWs layer for resisting the irreversible damage of the conductive network caused by skin deformation and electrode detachment. Compared to commercial Ag/AgCl gel electrodes, the BAP electrodes provide bioelectric signals of higher quality, especially under harsh interferences such as body movements and sweating. They impressively maintain stable signal throughout 7 days of continuous ECG monitoring, or during 10 cycles of electrode reusing. By virtue of the combined high signal/noise ratio and dynamic stability in surface EMG monitoring, the BAP electrodes also show advantages in finger motion recognition, and succeed in piano playing monitoring, including the recognition of five‐finger playing and music score, and the identification of articulation and dynamics.

## 2 Results and Discussion

### 2.1 The Design of the BAP Electrode

**Figure 1:**
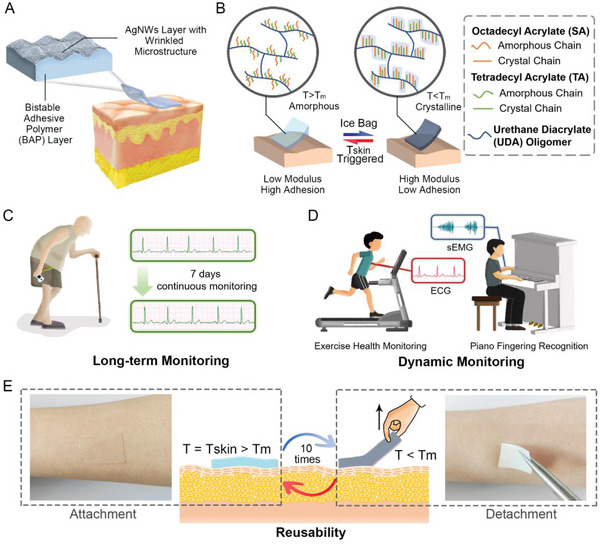
Schematic diagram of the working mechanism of BAP electrode and the advantages in bioelectric sensing. A) The schematic diagram of the BAP electrode structure. B) Schematic diagram of the on‐demand adhesion switch of BAP. The advantages of the BAP electrode in bioelectric signal sensing: C) long‐term monitoring for 7 days, D) dynamic monitoring, and E) reusability for ten times.

The BAP electrode consists of an AgNWs network with a wrinkled microstructure and a temperature‐sensitive BAP substrate (about 0.5 mm) (**Figure** **1**A). The preparation process is shown in Figure S1, Supporting Information. The BAP is formed by UV polymerization of octadecyl acrylate (SA), tetradecyl acrylate (TA), and urethane diacrylate (UDA) oligomer. The temperature response of BAP is attributed to the crystalline‐amorphous phase transformation of the side chains (SA and TA). UDA is a long‐chain oligomer that can form a bottlebrush polymer with the side chains. It is conducive to the ordered crystallization of the side chains, reduces the phase transition temperature range of the polymer, and improves the degree of crystallization of the side chains, making the change of modulus before and after the phase transition more significant.^[^ ^35^, ^38^ ^]^ Moreover, UDA homopolymer has a modulus of 0.827 MPa and an elongation at break of 1100%, which guarantees the low modulus and high ductility of BAP films.^[^ ^39^ ^]^ According to Figure 1B, when *T* > *T*_m_ (melting temperature), the polymer matrix becomes soft as the steric hindrance effect of side chains is diminished. As a result, the BAP exhibited high fluidity and dissipation characteristics, thus achieving low modulus and high adhesion. However, when *T* < *T*_m_, SA and TA transform into crystalline state, which stiffens the polymer chains, and the fluidity and dissipation characteristics are significantly reduced, resulting in the increase of modulus and the decrease of adhesion. Ideally, *T*_m_ should be slightly less than the skin temperature, so that BAP can not only quickly change into amorphous state merely through body heat triggering for conformal attachment of electrode on skin, but also transform instantly into crystalline state through cold water or ice bag treatment to achieve painless detachment and avoidance of electrode damage.

The combination of low modulus and high adhesion at skin temperature contributes to a conformal and stable skin‐electrode interface, which facilitates the maintenance of low interfacial contact impedance. The wrinkled microstructure of AgNWs percolation network endows the BAP electrode with stable conductivity against mechanical deformation. Therefore, the BAP electrode enables the bioelectric signal collection both in long‐term (Figure 1C) and dynamic (Figure 1D) conditions. The high modulus and low adhesion of BAP electrodes during detachment, as well as the excellent stretchability of the wrinkled AgNWs network, ensure the consistency of BAP electrode performance during multiple reuses (Figure 1E), which assists in reducing costs and electronic waste.

### 2.2 Mechanical and Adhesive Performance of BAP Electrodes

**Figure 2:**
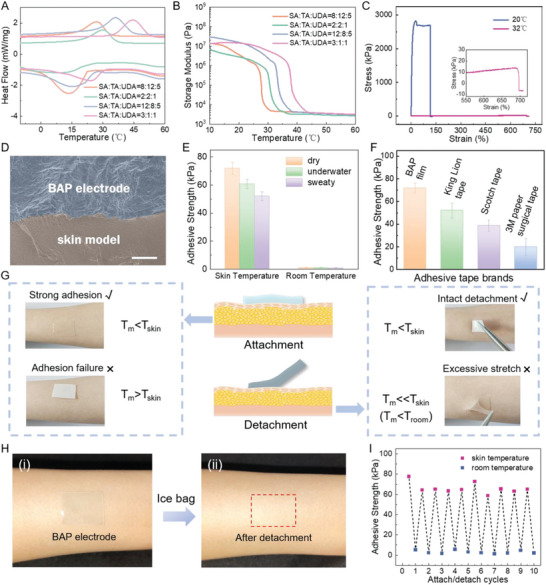
Mechanical and Adhesive Performance of BAP electrodes. A) DSC diagram of BAP with different composition ratios of SA: TA: UDA. B) Relationship between storage modulus and temperature of BAP with different composition ratios. C) Stress–strain curves of the BAP film at 20 and 32 °C. D) Cross‐sectional SEM image of the BAP electrode‐skin model at skin temperature; scale bar: 20 µm. E) Tensile adhesive strength of the BAP film on pig skin at skin temperature and room temperature in dry, underwater, and sweaty states. F) Comparison of tensile adhesive strength between the BAP film and commercial tapes on pig skin. G) Optical photographs of attachment (left) and detachment (right) of BAP with different *T*_m_. H) Optical photographs of skin when the BAP electrode was i) attached and ii) detached, with no skin redness or irritation observed. I) Tensile adhesive strength of the BAP electrode during 10 cycles of attachment.

As SA and TA have different melting points due to their different alkyl chain lengths, the copolymer BAP's phase transition temperature can be tuned to be slightly lower than skin temperature by adjusting the ratio of SA and TA in the polymer network. ​**Figure** **2**A exhibits the differential scanning calorimetry (DSC) curves of BAP with different ratios of SA, TA, and UDA. When the mass ratio of SA: TA: UDA is 2:2:1, *T*_m_ is about 30 °C, at which point the crystalline‐amorphous phase transformation can be activated simply by skin heat. Therefore, we chose the BAP with the ratio of 2:2:1 for the preparation of bioelectric electrode in the following study. The phase transition is further confirmed by the large variation of the storage modulus as a function of temperature (Figure 2B). The storage modulus of BAP decreased from 1682–2816 kPa in the crystalline state to 3.45–12.3 kPa in the amorphous state for the investigated recipes of BAP. At this point, the polymer stiffness drops significantly within 30 s due to the narrow phase transition temperature window located slightly lower than human body temperature. Furthermore, the higher fluidity of the BAP at the skin temperature gives it a higher elongation. As shown in Figure 2C, the elongation of BAP at break in the amorphous state (32 °C) is 697%, which is much larger than that in the crystalline state (20 °C, 116%).

The phase transition of BAP triggered by temperature also causes the change in adhesive properties. The high storage modulus at room temperature leads to significantly lowered adhesion strength between BAP films (with no AgNWs coating) and skin. On the contrary, the side chains in polymer networks change from crystalline to amorphous states above the phase transition temperature, which enhances the fluidity of the molecular chains and remarkably reduces the storage modulus, endowing BAP films with high energy dissipation characteristics, and improving the adhesive strength.^[^ ^40^ ^]^ The high fluidity also allows more adhesive functional groups such as amino‐ester bonds to be exposed to form sufficient interactions with the skin, further improving adhesion. In addition, the low‐modulus ensured conformal adhesion to skin without visible gaps, resulting in increased contact area (Figure 2D). The adhesive strength of BAP films to pig skin was as low as 0.90 ± 0.43 kPa at 20 °C (room temperature), then soared to 71.84 ± 4.40 kPa after being heated to 32 °C (skin temperature). At skin temperature, the adhesive strength of BAP films in dry state was 71.84 ± 4.40 kPa, while it decreased slightly in underwater and sweaty states, which were 60.83 ± 3.31 kPa and 52.01 ± 3.15 kPa. The adhesive strength of BAP films still maintains a high level (>50 kPa) even in sweaty state, enabling BAP films to adhere firmly to the skin (Figure 2E). This can be ascribed to the high hydrophobicity of the BAP, which hinders the infiltration of water into the interface between the BAP film and the target surface. Figure 2F discloses that our BAP substrate exhibits higher adhesive strength than several kinds of commercial adhesive tapes, which qualifies the BAP films as reliable adhesive tapes in practical applications. Adjusting the phase transition temperature of BAP to be slightly lower than the skin temperature enables a convenient switch of adhesive strength. For *T*_m_ higher than skin temperature, the body heat fails to trigger the high adhesion of BAP. For *T*_m_ much lower than skin temperature, the ice bag treatment is not able to provide a quick adhesion degradation to allow painless detachment and avoid electrode damage (Figure 2G). Importantly, after the AgNWs network transfer to BAP film, the obtained BAP electrode inherited high adhesive strength with no obvious decrease (Figure S2, Supporting Information). Due to the semi‐embedded structure of AgNWs at the polymer surface and the high fluidity of the substrate, the surface of the BAP electrode is still polymer‐dominated, which is conducive to the maintenance of adhesion strength. High adhesion ensures no relative displacement at the electrode‐skin interface during skin deformation (Figure S3, Supporting Information), which is of great significance for dynamic signal acquisition. After peeling off the electrodes from the skin, the texture of the skin can be completely reproduced, indicating the intimate topographic matching at the skin surface (Figure S4, Supporting Information). Although the pressure‐sensitive adhesive layer of the commercial Ag/AgCl electrode also has high adhesive strength to skin, it will induce skin irritation and discomfort (Figure S5, Supporting Information). The adhesion strength of the electrode decreased greatly after cold water or ice bag treatment, which facilitated easy removal from the skin without causing any pain, irritation, or skin redness. (Figure 2H). What's more, after 10 cycles of attaching and peeling‐off, the adhesive strength of the BAP electrode did not go through appreciable degradation, still providing a high level of around 60 kPa (Figure 2I) for potential multiple reuses.

### 2.3 Electrical Performance of BAP Electrodes

**Figure 3:**
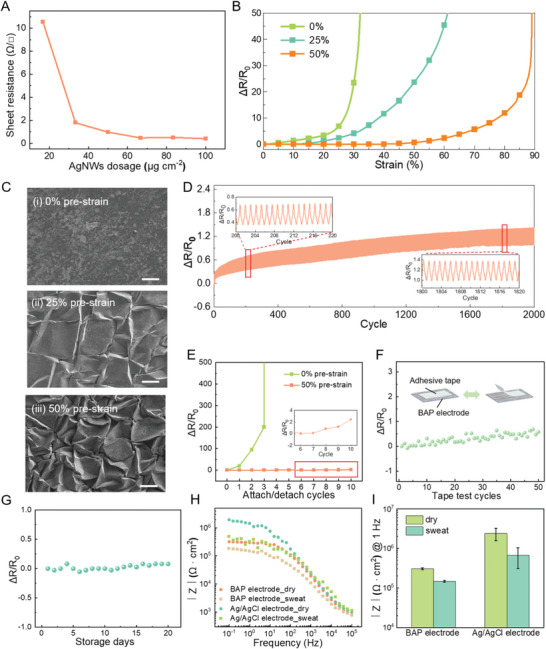
Electrical Performance of BAP electrodes. A) Relationship between AgNWs dosage and electrode sheet resistance. B) The relative resistance variation versus stretch strain of BAP electrodes with different pre‐strains (0%, 25%, and 50%). C) SEM images of BAP electrodes surface with different pre‐strains i) 0%, ii) 25%, iii) 50%, scale bar: 200 µm. D) The relative resistance variation of the BAP electrode during 2000 cycles of stretching at strain of 40%. E) The relative resistance variation of BAP electrodes (pre‐strains of 0% and 50%) during 10 consecutive attach/detach cycles. F) The relative resistance variation of BAP electrode during 50 cycles of tape test. G) The relative resistance variation of BAP electrode during 20 days storage in air. H) On‐skin interface impedance of the BAP electrode and Ag/AgCl electrode under dry and sweaty conditions. I) Skin‐electrode interface impedance of the BAP electrode and Ag/AgCl electrode under dry and sweaty conditions at 1 Hz.

Besides reliable interface adhesion, the high conductivity of the electrode is another key factor to guarantee the acquisition of high‐quality bioelectric signals. AgNWs‐based percolation network was used as the conductive layer in this work considering the high conductivity, good flexibility, and stretchability.^[^ ^41^, ^42^ ^]^ **Figure** **3**A presents the relationship between the sheet resistance of BAP electrodes and the dosage of AgNWs. The sheet resistance of BAP electrodes decreased with increasing AgNWs dosage, and it reached as low as 0.473 Ω □^−1^ at 67 µg cm^−2^. As the dosage continues to increase, the electrode conductivity leveled off, and the excessive amounts of AgNWs also increased the risk of exfoliation. Therefore, the optimized amount of AgNWs in this work was set at 67 µg cm^−2^. The DSC diagram of the BAP film and the BAP electrode shows that AgNWs covering the surface of the BAP substrate do not significantly affect the behavior of the BAP phase transition (Figure S6, Supporting Information).

In order to endow the AgNW‐based conductive network with high tolerance to stretch caused by skin deformation (generally less than 50%) in daily activities, a biaxial pre‐straining strategy was adopted to construct wrinkled microstructure in the conductive layer. Figure S7, Supporting Information shows the optical microscopy photographs of the BAP electrode at 20 and 32 °C, which confirms that the wrinkled microstructure is well maintained in the amorphous and crystalline states. Uniaxial pre‐straining leads to a large number of cracks perpendicular to the pre‐straining direction in the conductive network due to Poisson effect (Figure S8, Supporting Information). By contrast, the wrinkled electrode of biaxial stretching maintains the integrity of the conductive network of AgNWs and better resists multi‐directional skin strain. The electrode obtained by biaxial pre‐straining has low resistance (8.9 Ω), which ensures the high conductivity of the electrode, while the electrode obtained by uniaxial pre‐straining has high resistance (1042.6 Ω) (Figure S9, Supporting Information). Moreover, the pre‐straining degree of BAP electrodes also has a significant effect on the ability of the conductive layer to resist deformation. Figure 3B shows the tensile strain dependence of relative resistance change of BAP electrodes with different pre‐straining degrees. The resistance of the 0%, 25%, and 50% pre‐straining electrodes started to dramatically increase (Δ*R/R*_0_ > 5) under 25%, 35%, and 70% tensile strain, while the conductive network was considerably damaged (Δ*R/R*_0_ > 50) under 30%, 60%, and 90% tensile strain. At 50% tensile strain, the Δ*R/R*_0_ of the BAP electrode was only 0.6 when the pre‐strain was 50%, while the Δ*R/R*_0_ of the BAP electrode was 23.61 when the pre‐strain was 25%, and the electrode with pre‐strain of 0% lost its conductivity. Higher pre‐strain results in higher wrinkle density, which can store larger strain along the pre‐strain direction (Figure 3C). These wrinkles gradually flatten and release stress when the electrode is subjected to tensile forces, which inhibits crack generation and imparts stable conductivity during the electrode deformation. Figure S10, Supporting Information illustrates SEM diagrams of the BAP electrode with different pre‐straining ratios at 50% tensile strain. There were numerous penetrating fractures in the 0% pre‐straining electrode, while a few penetrating cracks in the 25% pre‐straining electrode. In the 50% pre‐straining electrode, only the wrinkled microstructure was partly flattened, and no penetrating fractures were discovered. Therefore, the BAP electrode with 50% biaxial pre‐strain was adopted in our work unless otherwise specified. Because of the low initial resistance of the BAP electrode (1.79 Ω), the resistance was still kept at a low level of 3.56 Ω (Δ*R/R*_0_ = 0.98) after 2000 cycles of stretching, which can meet the needs of bioelectric monitoring (Figure 3D). After cyclic stretching, the wrinkled structure is well maintained and there is no observable network disconnection in the AgNWs network (Figure S11, Supporting Information). The resistance of the electrode with wrinkled microstructure formed by 50% pre‐straining was kept stable when the electrode was attached and detached from the skin for 10 cycles, while the electrode without pre‐straining underwent a sharp rise of resistance merely after 3 cycles (Figure 3E). Therefore, the wrinkled microstructure also favors the reusability of BAP electrodes. SEM image proves no obvious damage to the conductive network of the BAP electrode after 10 cycles of repeated use (Figure S12, Supporting Information). In addition, the high fluidity of the BAP substrate activated at high temperature leads to the configuration of AgNWs network as being semi‐embedded in the polymer substrate, which anchors the conductive layer for enhanced stability (Figure S13, Supporting Information). In tape test, the Δ*R/R*_0_ of the BAP electrode was only 0.5 after the electrode was pasted with tape for 50 times (Figure 3F). After 20 days storage in air, resistance increase of the BAP electrode was negligible, testifying its excellent environmental stability (Figure 3G). The dynamic and static stability of the conductance provides an important basis for long‐term stable bioelectric monitoring of BAP electrodes.

Low skin‐electrode contact impedance is another guarantee for the high quality of bioelectric signals. Figure 3H indicated that the skin‐electrode contact impedance of the BAP electrodes was lower than that of the Ag/AgCl electrodes at all testing frequencies in both dry and sweat states. Especially in the low frequency range wherein the bioelectric signal is located, the contact impedance of the BAP electrodes has a significant advantage. For example, as shown in Figure 3I, the skin‐electrode contact impedance of the commercial Ag/AgCl electrodes was 2410.61 ± 842.54 kΩ cm^2^ at 1 Hz in dry state, while that of the BAP electrodes was 304.71 ± 18.02 kΩ cm^2^, which was only about one‐eighth of that of the commercial electrodes. In a sweaty state, the contact impedance of both kinds of electrodes decreased. Even though, the contact impedance of the BAP electrodes (147.70 ± 8.69 kΩ cm^2^ @ 1 Hz) was still much smaller than that of commercial Ag/AgCl electrodes (673.93 ± 367.42 kΩ cm^2^ @ 1 Hz). In addition, when the electrodes were attached to the sweaty and moving arm, the standard deviation of contact impedance of the BAP electrodes at 1 Hz was only 8.69 kΩ cm^2^, which was much smaller than that of the commercial Ag/AgCl electrodes (367.42 kΩ cm^2^), demonstrating the superb stability of the BAP electrodes under dynamic conditions. In general, we attribute the low and stable skin‐electrode contact impedance under both static and dynamic (moving or sweating) conditions to the remarkable combination of high conductivity, low modulus, and high adhesion of the BAP electrode triggered by skin temperature. Consequently, they demonstrate great application potential in the acquisition of bioelectric signals in various daily activities.

### 2.4 Bioelectric Signals Monitoring Using BAP Electrodes

#### 2.4.1 Monitoring of ECG Signals

**Figure 4:**
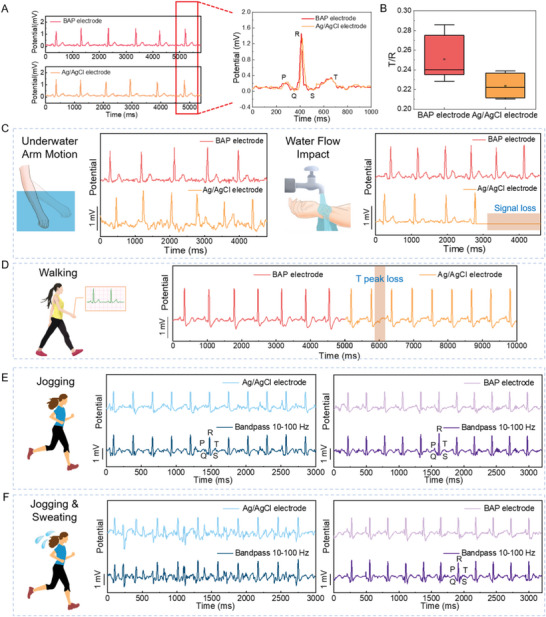
BAP electrodes for ECG signal monitoring in static and dynamic states. A) ECG signals and B) T/R values recorded by BAP and Ag/AgCl electrodes in static state. C) ECG signals recorded by BAP electrodes and Ag/AgCl electrodes during arm motion underwater (left) and water flow impact (right) underwater conditions. D) ECG signals recorded by BAP electrodes and Ag/AgCl electrodes during walking. ECG signals recorded by BAP electrodes and Ag/AgCl electrodes during E) jogging and F) jogging with simulated sweat.

Heart disease is characterized by abruptness, intermittency, and permanence, and patients require long‐term continuous treatment and monitoring. Hence, long‐term ECG monitoring is of great clinical significance. It requires electrodes to maintain both high accuracy and robust stability during signal acquisition in various daily situations. **Figure** **4**A compares the ECG signals recorded by BAP electrodes and Ag/AgCl gel electrodes in static state. Both waveforms showed clear P‐QRS‐T peaks. The sensitivity of ECG electrodes can evaluate the quality of ECG signals, which is defined as the relative voltage ratio of the measured T peak to R peak (T/R value). A higher T/R value indicates a higher accuracy of ECG signals. The sensitivity of the BAP electrodes was 0.25, higher than that of the Ag/AgCl electrodes (0.22) during static ECG acquisition (Figure 4B). Therefore, the BAP electrode promises more accurate signals for clinical heart disease diagnosis.

In daily ECG monitoring, various activities and complex conditions challenge the dynamic stability of the signal acquisition. Figure 4C compares the underwater ECG signals of BAP electrodes and Ag/AgCl electrodes. For underwater arm motion and water flow impact, the BAP electrode could acquire stable ECG signals with clear P‐QRS‐T peaks, which verified their application in bioelectric monitoring during dynamic underwater activities such as swimming and bathing. By contrast, for Ag/AgCl electrodes, the P and T peaks of ECG signals attenuated or even disappeared when swinging the arm underwater. The impact of water flow even brought about the delamination of the Ag/AgCl electrode from the skin and the interruption of ECG signals. In addition to underwater environments, BAP electrodes can offer significant advantages in body‐moving situations such as walking or jogging. During walking, the P‐QRS‐T peaks of ECG signals acquired with the BAP electrode were distinguishable, whereas the T peaks of the ECG signal measured with the Ag/AgCl electrode failed to be distinguished due to interference induced by motion artifact (Figure 4D). During jogging, the acquisition of ECG signals was violently disturbed by the shaking of the test box and conducting wires, so that P and T peaks were completely submerged in strong noise. Therefore, the raw signals were band‐pass filtered at 10 Hz – 100 Hz. After filtering, Figure 4E shows the P and T peaks of the ECG signals measured by the BAP and commercial Ag/AgCl electrodes can be recognized clearly. Body movement is usually accompanied by sweating, another main factor that causes signal interference. Here, simulated sweat (normal saline) was used to evaluate the anti‐sweat interference ability of the two electrodes. When soaked in simulated sweat, the ECG signals of the Ag/AgCl electrodes underwent severe distortion, and the P and T peaks were difficult to be differentiated even after band‐pass filtering. In sharp contrast, the signals acquired by the BAP electrodes were almost unaffected and still afforded a clear P‐QRS‐T waveform (Figure 4F). The comprehensive performance comparison under various conditions verified the advantage of our BAP electrodes over commercial gel electrodes for daily ECG monitoring, especially when it comes to challenging outdoor activities.

**Figure 5:**
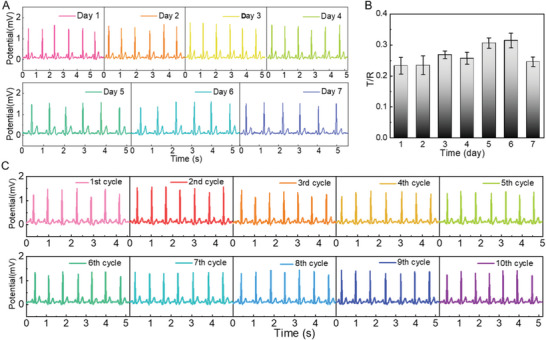
BAP electrodes for long‐term and reusable ECG signal monitoring. A) ECG signals and B) T/R values monitored continuously by BAP electrodes for 7 days. C) ECG signals monitored by BAP electrodes during 10 consecutive attach/detach cycles.

Remarkably, the long‐term electrical and adhesive stability of the BAP electrodes collectively enable long‐term and continuous signal monitoring. During the 7‐day ECG monitoring test, the BAP electrodes did not experience detachment or damage during daily activities, and the ECG signals remained stable throughout, without baseline drift or peak loss (**Figure** **5**A). Figure 5B presents the sensitivity of the ECG signal on each day, which maintains a high level of around 0.25 and a low relative standard deviation of only 11.5%. Simultaneously, the non‐solvent dry nature of the BAP electrode exempts itself from the risk of water loss, making it superior to gelled electrodes in long‐term use.

Long‐term monitoring of bioelectrical signals requires good biocompatibility of electrodes. Figure S14, Supporting Information shows the live/dead fluorescence staining images of NIH3T3 cells after 24 h of culturing. There is no significant difference between the cells cultured with and without BAP electrodes, indicating that BAP electrodes are not cytotoxic. Moreover, the BAP electrode was attached to the skin for 7 days, and there was no skin redness or allergy after removing it (Figure S15, Supporting Information). Therefore, the BAP electrode has good biocompatibility and anti‐allergenic properties in long‐term monitoring.

Moreover, after ice bag treatment, the crystalline‐state BAP electrode exhibited not only a considerably decreased skin adhesive strength, but also a relatively stiffer conductive layer. Both of these effects effectively suppress the otherwise excessive stretch/distortion on BAP electrodes which could damage the conductive network. As a result, BAP electrodes allow multiple reuses, which contribute to reducing costs and electronic waste. As shown in Figure 5C, the ECG signals recorded by BAP electrodes were stable after repeated use on the skin for ten times. High retention of mechanical properties and electrical conductivity during cyclic adhesion and debonding is responsible for such robust reusability.

#### 2.4.2 Monitoring of surface EMG Signals

**Figure 6:**
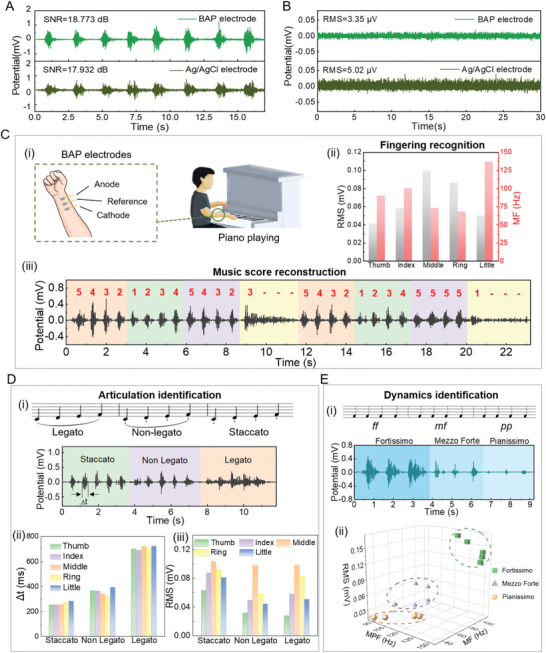
BAP electrodes for sEMG signal monitoring. A) The sEMG signals and SNR value of BAP electrodes and Ag/AgCl electrodes in fist clenching. B) The sEMG signal baseline and RMS value of BAP electrodes and Ag/AgCl electrodes. C) i) Schematic diagram of BAP electrodes in piano fingering identification. ii) RMS and MF of the sEMG signals of each finger during playing. iii) The sEMG signals and the recognized music score monitored by BAP electrodes. D) i) The sEMG signals collected under different types of piano articulation and ii) Δ*t* and iii) RMS of sEMG when playing piano with each finger. E) i) The sEMG signals at different music dynamics, ii) statistical distribution results of RMS, MPF, and MF extracted from the sEMG signals corresponding to different music dynamics (pianissimo, mezzo forte, and fortissimo).

Surface EMG (sEMG) records the electrical activity of muscles from the surface of the skin, which is non‐invasive and easy to operate, and has broad prospects in the field of human‐machine interaction.^[^ ^43^, ^44^ ^]^ Precise motion recognition requires the signal accuracy to be as high as possible. The sEMG signals of BAP electrodes and Ag/AgCl gel electrodes were recorded during fist clenching (**Figure** **6**A). The SNR of the sEMG signals recorded by the BAP electrodes was 18.773 dB, which was higher than that recorded by the Ag/AgCl gel electrodes (17.932 dB). The baseline potential represents the noise level, which can characterize the signal quality from another aspect. As shown in Figure 6B, the root mean square (RMS) of the baseline potential recorded by the BAP electrodes was only 3.35 µV, which was lower than that recorded by the Ag/AgCl gel electrodes (5.02 µV). Both high SNR and low baseline potential represent better quality of sEMG signals recorded by BAP electrodes. Therefore, the BAP electrodes are more promising to implement sEMG‐based human motion recognition with high precision. Piano playing is selected here as a representative application for it demands delicate finger movements.

For piano‐learning beginners, finger movement recognition can assist in efficient practice. Although strain sensors attached to the finger can give high recognition accuracy, they inevitably limit finger movements and affect proprioception during practice. The BAP electrodes attached to the arm to collect sEMG signals can avoid such interference to the piano playing. The BAP electrodes were placed in the position shown in Figure 6C‐i for fingering recognition. When playing the piano, the thumb, index finger, middle finger, ring finger, and little finger exclusively correspond to five notes, do (1), re (2), mi (3), fa (4) and so (5) respectively. The original signals of sEMG are complex and difficult to compare directly. Therefore, we need to analyze the sEMG signals in the time domain and the frequency domain to more intuitively and accurately show the signal differences of different fingers. We selected RMS as the time domain eigenvalue, which represents the amplitude of the sEMG signal and reflects the degree of muscle activation. The median frequency (MF) is an eigenvalue in the frequency domain, which represents the speed of muscle contraction. Compared with other frequency‐domain eigenvalues, MF has stronger anti‐noise performance.^[^ ^45^ ^]^ According to Figure 6C‐ii, we extracted the RMS and MF values of sEMG signals when five fingers played, so as to realize the recognition of different playing fingers. Thus, we can reconstruct the music score by identifying the playing fingers from the sEMG signals (Figure 6C‐iii). Further, for different piano articulations (the specific way to strike a piano key to make a desired sound), there are significant differences in the duration of sEMG signals (Δ*t*), as shown in Figure 6D‐i. Under the same playing rhythm, the Δ*t* produced by staccato is the shortest, followed by non‐legato, and legato is the longest, as shown in Figure 6D‐ii. In this way, the sEMG signals measured by BAP electrodes can distinguish different types of piano articulation, including staccato, non‐legato, and legato. Also, Figure 6D‐iii indicates that the RMS values of the sEMG signals of the five fingers are significantly different when playing the piano under staccato, non‐legato, and legato. Consequently, the recognition of the five fingers can be realized under different piano articulations. Different playing rhythms can also be identified through the intervals of the sEMG signals (Figure S16‐i, Supporting Information). Due to the instantaneity of sEMG signal acquisition, different sEMG intervals represent different playing rhythms. The RMS values of the sEMG signals generated by five‐finger playing still remain significantly different in different playing rhythms, thus it still has strong discriminability for five fingers (Figure S16‐ii, Supporting Information). In addition, the sEMG signal can also identify different music dynamics (defined as pianissimo, mezzo forte, and fortissimo). During the experiment, the piano player maintained the playing gesture, hitting the piano key with only one finger while the other fingers remained relaxed. In this case, only the muscles corresponding to the playing finger produce sEMG signals, and the signals corresponding to the other fingers are ignored. Different music dynamics of the ring finger were chosen as examples in Figure 6E‐i. When RMS, MF and mean power frequency (MPF) feature values were selected in the time domain features, it can be seen that the feature sets corresponding to pianissimo, mezzo forte, and fortissimo are located in distinct zones in the triaxial coordinate system (Figure 6E‐ii).

## 3 Conclusion

In summary, this study proposed a temperature‐triggered bioelectric electrode with on‐demand adhesion and modulus based on BAP and AgNWs networks. The electrode achieved high adhesion to the skin and painless detachment simultaneously. Beyond this, ten times of reuse and long‐term ECG monitoring for 7 days without interference from human movements, sweating, and bathing were also realized. Compared with the commercial Ag/AgCl gel electrode, the BAP electrode has a higher SNR (18.773 dB) and a lower baseline noise (3.35 µV) in sEMG monitoring, which enable them to facilitate smart training on piano playing. These results demonstrate the great potential of BAP electrodes in the fields of long‐duration/dynamic health monitoring and diagnostics, and high‐precision human‐computer interactions such as prosthetic manipulation and emotion recognition.

## 4 Experimental Section

### Materials

SA (Sigma Aldrich), TA (TCI), and DMPA (Sigma Aldrich) were purchased from Greagent (Shanghai Titan Scientific Co., Ltd.). UDA oligomer (CN9021NS, Sartomer) was obtained from Chemelite Inc. The AgNWs solution (CST‐NW‐S70, 10 mg mL^−1^, average diameter of 70 nm, length of 30 ± 5 µm, solvent was IPA) was purchased through XFNANO Corporation. Ag/AgCl gel electrodes (CH50RB) were provided by Heal Force Bio‐meditech Holdings Limited.

### Preparation of BAP Films

SA, TA, and UDA were mixed in the ratio of 2:2:1. 0.5 wt% DMPA was added to the mixture as a photoinitiator. The mixed solution was treated with ultrasound at 40 °C for 1 h to make it evenly mixed. Remove bubbles from the mixed solution in a vacuum drying oven. BAP films were obtained by pouring the mixed solution into the mold and UV curing for 3 min.

### Fabrication of BAP Electrodes

The AgNWs solution was diluted with alcohol, and the AgNWs network was obtained on a PTFE membrane by suction filtration. The BAP film was pasted in the center of an Ecoflex film. The Ecoflex film was biaxially stretched, and the BAP film was driven to produce 50% pre‐straining. The AgNWs network on the PTFE membrane was transferred to the BAP film, and the BAP electrode with wrinkled microstructure was obtained after unloading. The process of pre‐straining and unloading was completed at 40 °C. The BAP electrode was connected to the external circuit with copper conductive tape, and silver paste was coated on the interface between the two to ensure the circuit ran.

### Characterization and Electrophysiological Signal Monitoring

The crystalline‐amorphous phase transformation temperature of polymer was measured with a differential scanning calorimeter (DSC200F3, Netzsch, Germany) in the range of −25 to 70 °C at a rate of 15 °C min^−1^. The temperature–storage modulus curves of polymer were characterized by a rheometer (Anton Paar, MCR301, Germany) using a temperature‐scanning oscillation mode with a temperature range of 0 to 60 °C, a heating rate of 1.8 °C min^−1^, a frequency of 1 Hz, and a strain of 1%. The universal tensile testing machine (CMT6103, MTS Systems, China) was used to test the stress–strain curves of BAP films at a tensile rate of 0.3 mm s^−1^ and a standard distance of 15 mm. The tensile adhesive strength was measured by the force gauge (MARK‐10, USA), and the moving rate of the force gauge was 500 mm min^−1^. The tensile adhesion strength was determined by the following formula: *P = ‐F/S*, where *F* is the peak force (negative value) read by the force gauge during movement and *S* is the area of the BAP film or tape. The field emission scanning electron microscope (SU8200, Hitachi, Japan) was used to characterize the surface structure of the BAP electrode and its interface with the pig skin model. The pigskin model was prepared by PDMS using the reverse mold method.

A low impedance surface impedance instrument (MCP‐T360, Japan) was used to measure the sheet resistance of the electrodes with different dosages of AgNWs. The electrochemical station (Autolab, Germany) was used to analyze the electrode resistance changes with strain, the cycling stability of electrode resistance, and the skin‐electrode contact impedance spectrum. In the cyclic stability test, the standard distance was set at 5 cm, the stretch‐recovery rate was 0.1 mm s^−1^, and the test temperature was 32 °C. During the skin‐electrode contact impedance test, the electrodes were attached to the skin of the forearm, the electrode spacing was 4 cm, the voltage was 0.01 V, and the frequency range was 0.1 Hz–10^5^ Hz. The long‐term electrical stability of the electrode and the interface stability between the conductive network and the substrate were tested by a multimeter (FLUKE 18B+, China).

The ECG signals were recorded by an ECG monitor (Heal Force PC‐80B, China), and the electrodes were attached to the left wrist, right wrist, and the inside of the left ankle. The sEMG signals were monitored by a surface EMG collector (ZJE‐II, China) at a sampling frequency of 1000 Hz. Matlab 2018a and Origin 2020 software were used to process the ECG and sEMG signals. The skin was wiped with alcohol before the electrodes were attached.

In vitro cell experiments were performed as follows: the 10 mm diameter BAP electrodes were sterilized by high temperature and pressure steam (121 °C for 20 min) and placed in a 24‐well plate, and NIH3T3 cells were seeded on the surface of the materials. The cells were incubated for 24 h at 37 °C in a 5% CO2 incubator. Cells in 24‐well plates were washed with PBS to remove excess serum. The staining solution (BestBio, BB‐4126) was added for cell staining, and the cells were incubated at room temperature in the dark for 15 min. Then the cells were observed and photographed using a fluorescence microscope (Zeiss, Axio Vert.A1).

## Conflict of Interest

The authors declare no conflict of interest.

## Supporting information

Supporting Information

## Data Availability

The data that support the findings of this study are available from the corresponding author upon reasonable request.
