# Supplementary material for: Temperature‐Triggered Adhesive Bioelectric Electrodes with Long‐Term Dynamic Stability and Reusability
Source: Adv Sci (Weinh). 2023 May 18;10(22):2300793. doi: 10.1002/advs.202300793 (PMC10401176; doi:10.1002/advs.202300793)
Supplement: Supplementary file 1 — Supporting Information [file ADVS-10-2300793-s001.pdf]

ADVANCED SCIENCE
Open Access

Supporting Information

for *Adv. Sci.*, DOI 10.1002/advs.202300793

Temperature-Triggered Adhesive Bioelectric Electrodes with Long-Term Dynamic Stability and Reusability

Huiting Lai, Yan Liu, Yin Cheng, Liangjing Shi, Ranran Wang* and Jing Sun*

Supporting Information

Temperature-triggered adhesive bioelectric electrodes with long-term dynamic stability and reusability

Huiting Lai, Yan Liu, Yin Cheng, Liangjing Shi, Ranran Wang*, and Jing Sun*

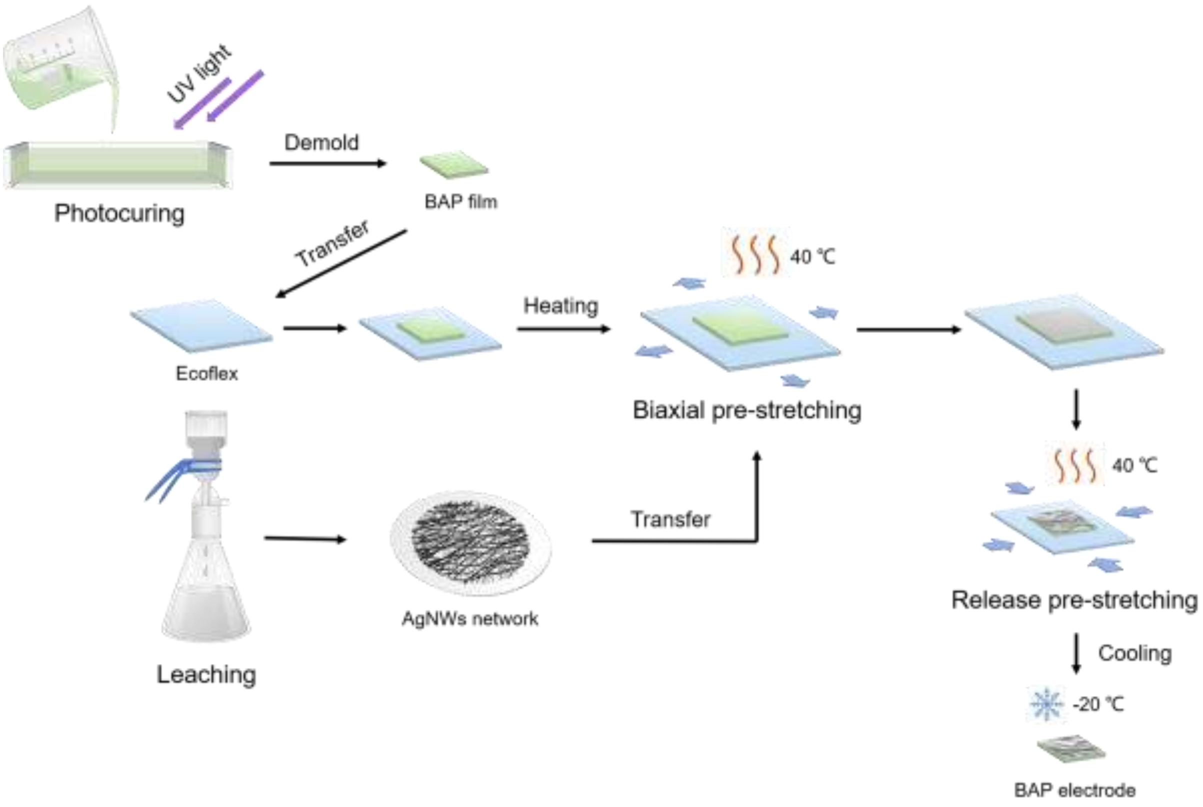

Figure S1. The flow chart of BAP electrode preparation.

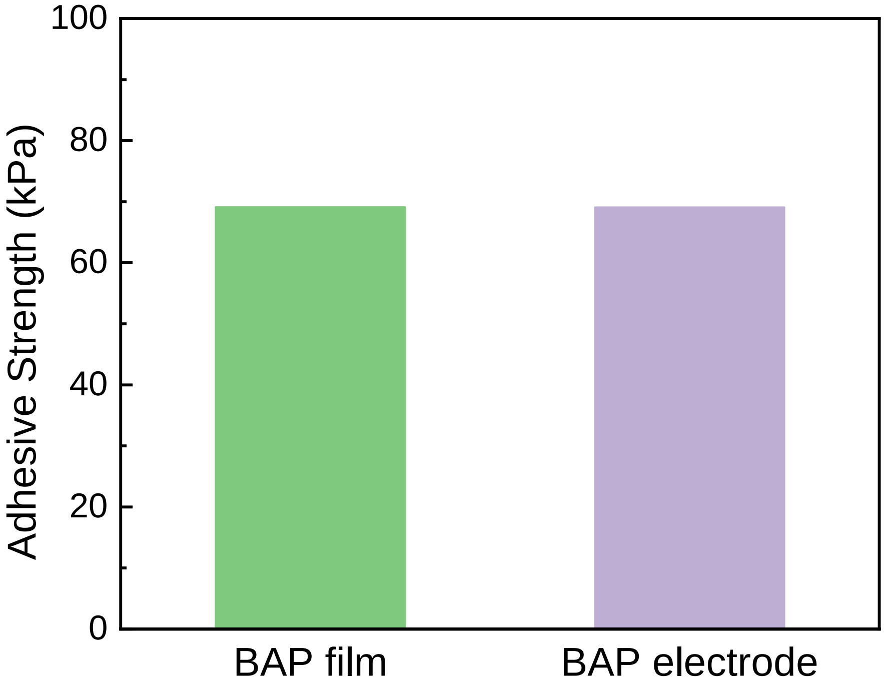

Figure S2. Tensile adhesive strength of the BAP electrode and the BAP film.

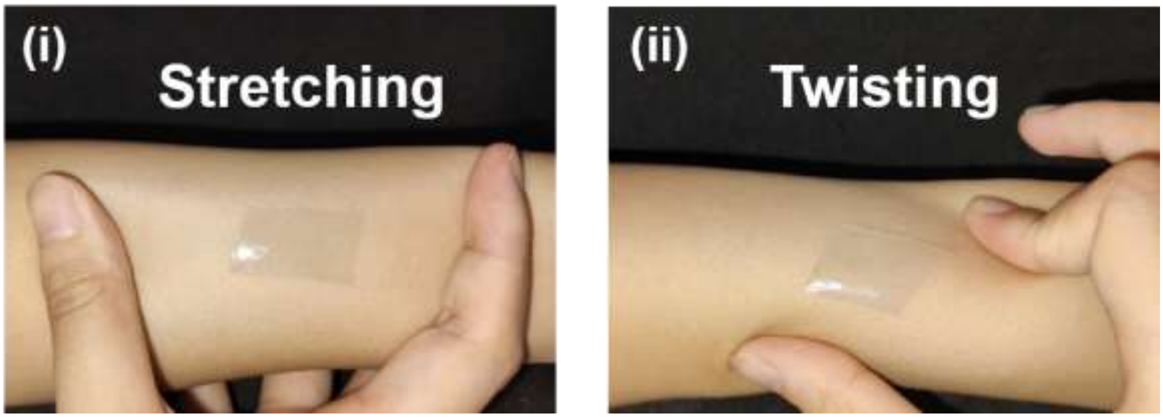

Figure S3. Optical photographs of the BAP electrode attached to the skin when being (i) stretched and (ii) twisted.

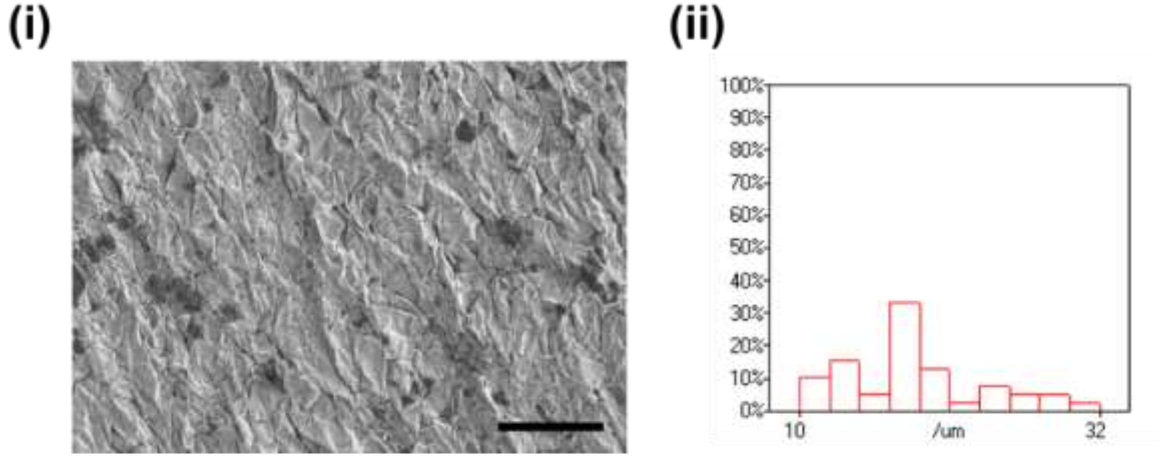

Figure S4. (i) SEM diagram and (ii) size distribution of skin texture reproduced by the BAP electrode. Scale bar: 200 μm.

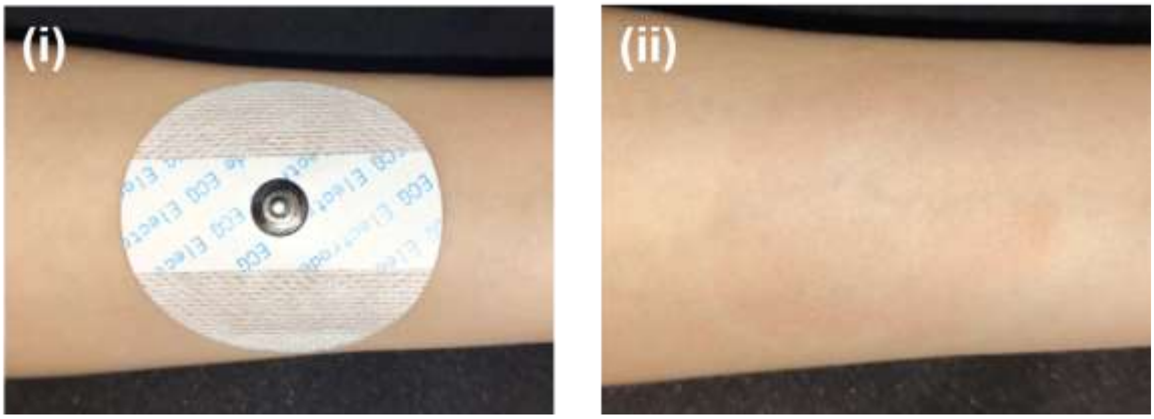

Figure S5. Optical photographs of skin when Ag/AgCl gel electrode being (i) attached and (ii) detached. Obviously, the long-duration wearing of commercial gel electrode caused skin redness.

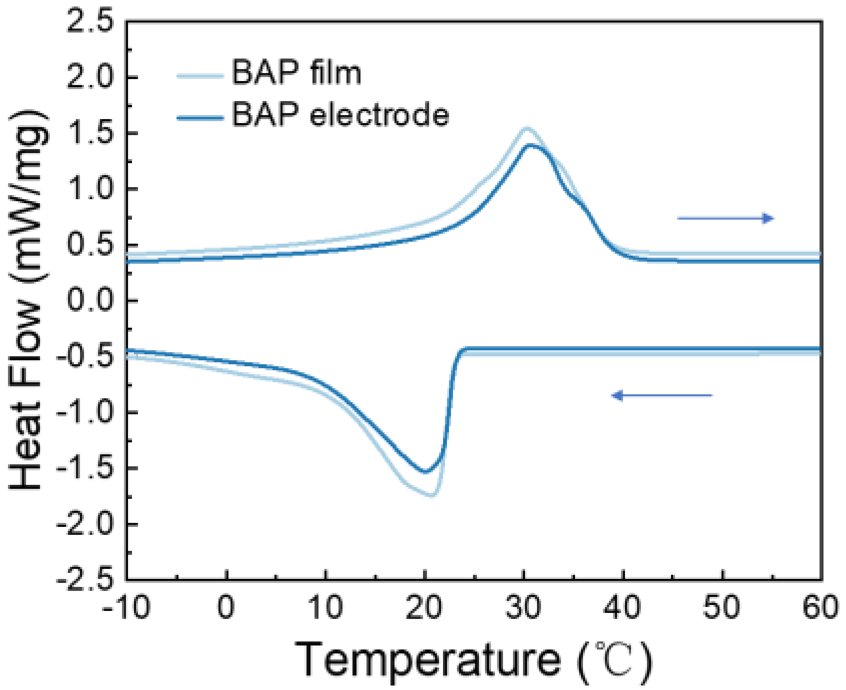

Figure S6. DSC diagram of BAP film and BAP electrode.

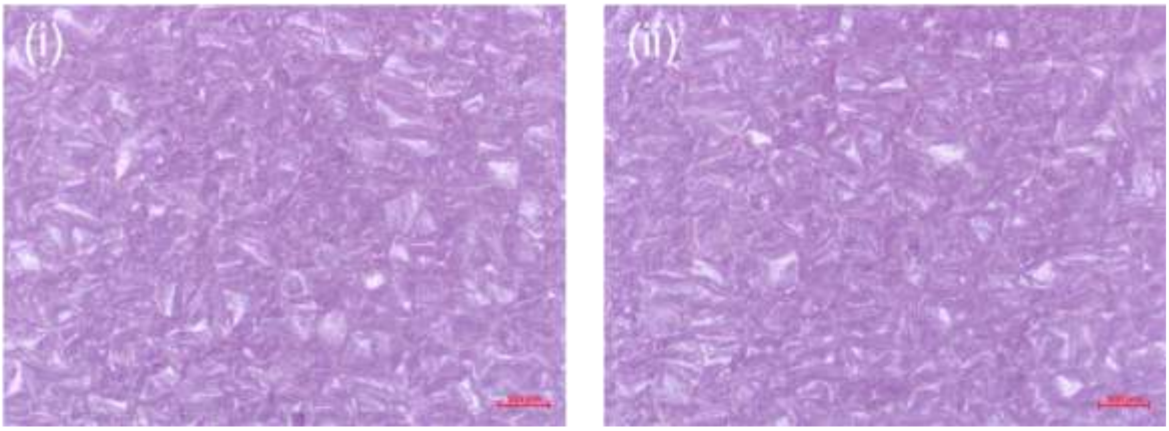

Figure S7. Optical microscopy photographs of the BAP electrode at (i) 20 ^{o}C and (ii) 32 ^{o}C.

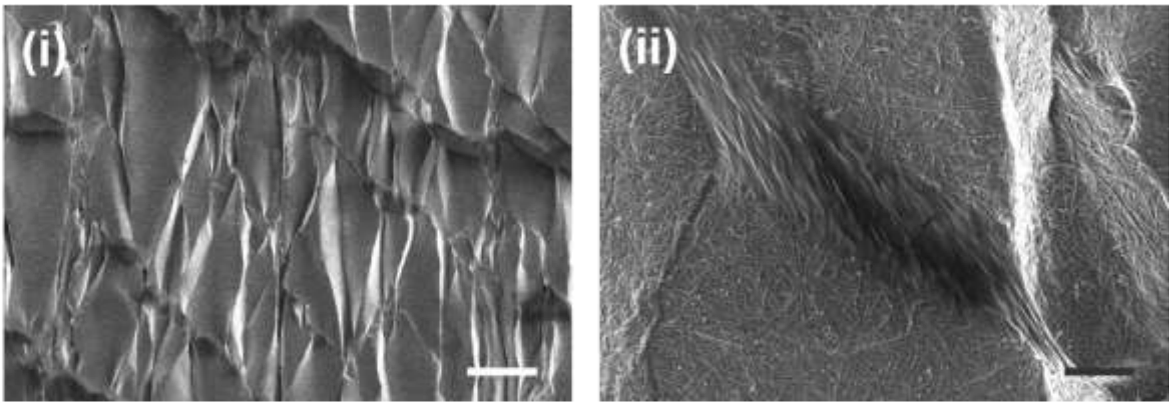

Figure S8. SEM diagrams of the BAP electrode by uniaxial pre-straining. Scale bar: (i) 200 μm; (ii) 20 μm.

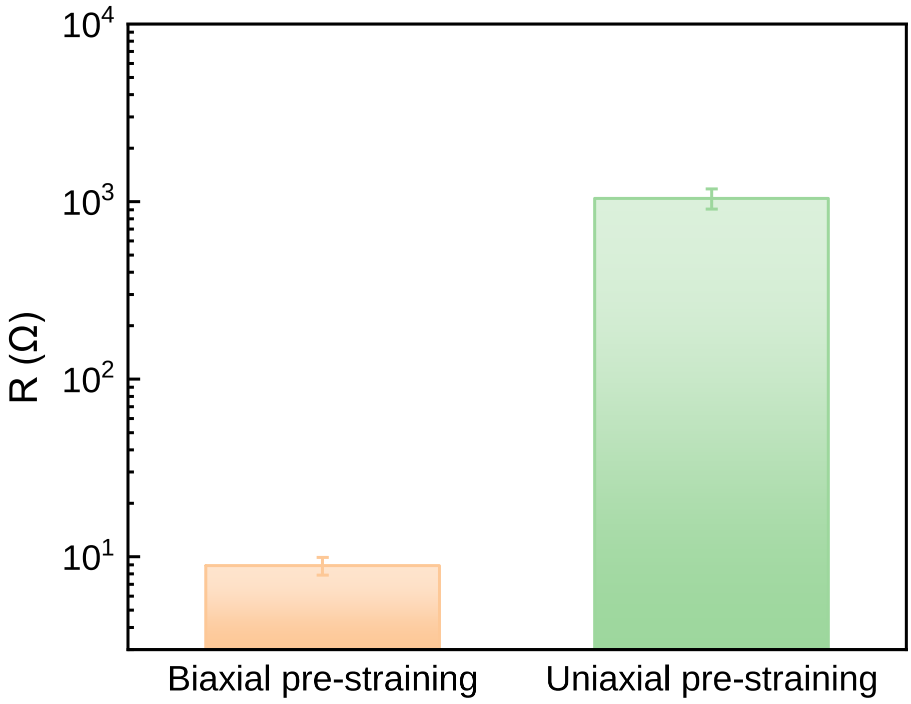

Figure S9. Resistance of BAP electrodes with uniaxial pre-straining and biaxial pre-straining.

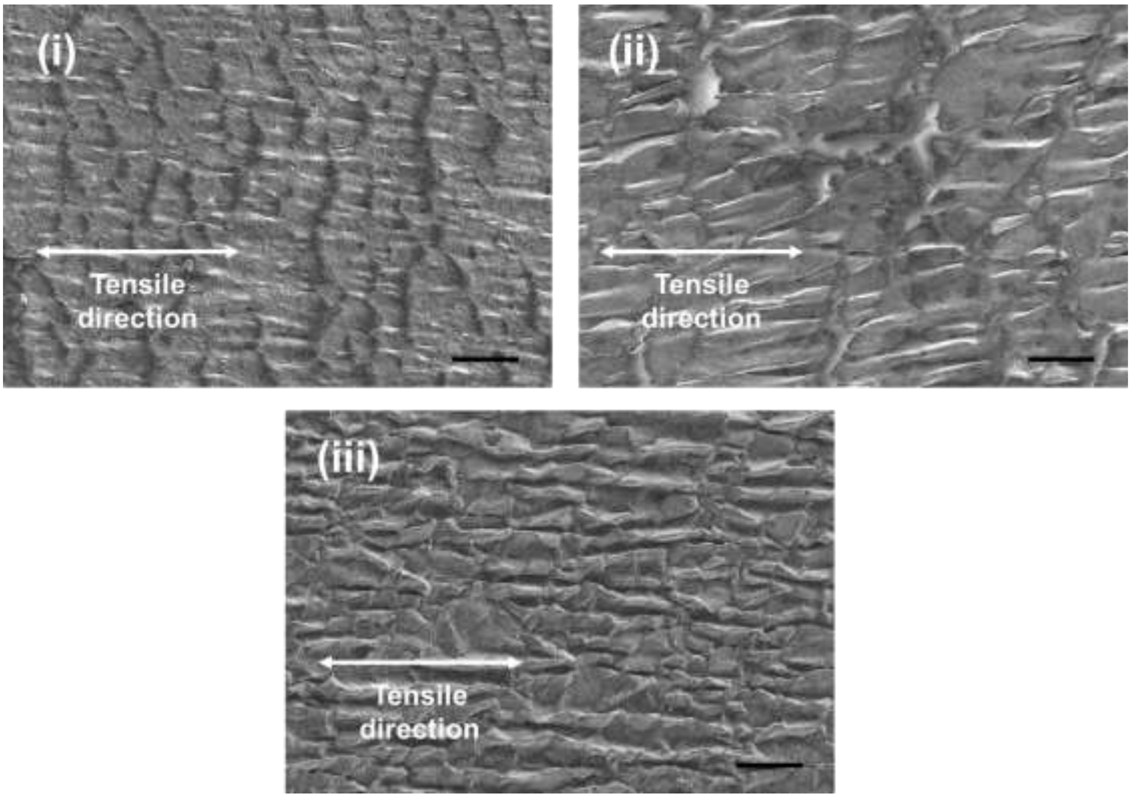

Figure S10. SEM diagrams of the BAP electrode with different pre-straining ratios at 50% tensile strain (i) 0%; (ii) 25%; (iii) 50%. Scale bar: 200 μm.

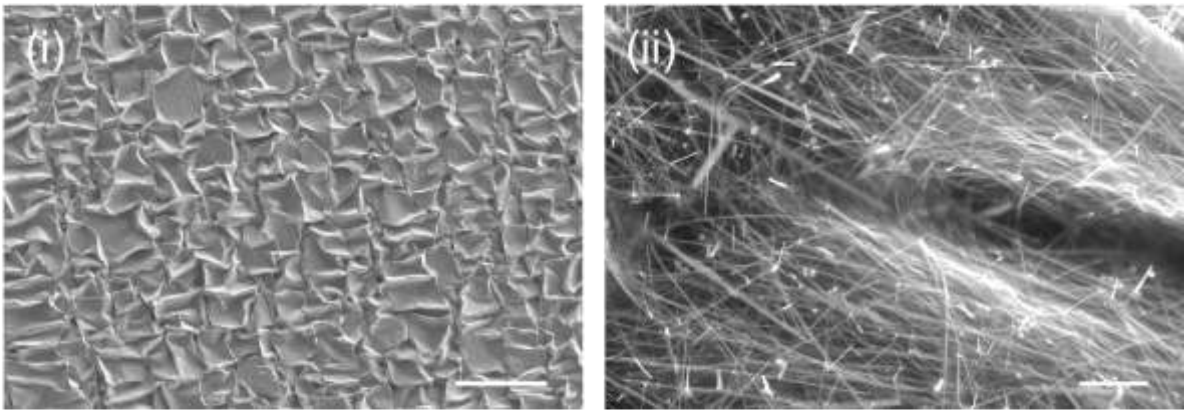

Figure S11. SEM diagrams of the BAP electrode after 2000 cycles of stretching at strain of 40%. Scale bar: (i) 200 μm; (ii) 5 μm.

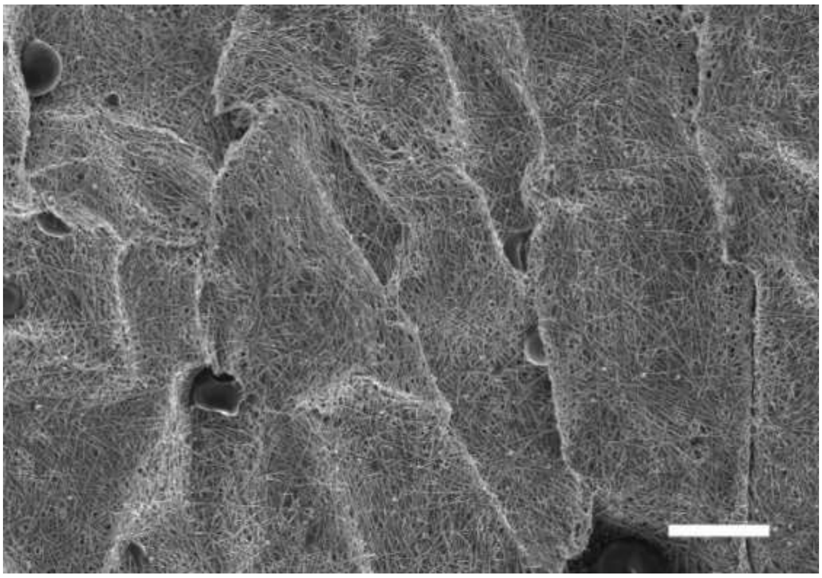

Figure S12. SEM diagram of the BAP electrode after being repeatedly attached to skin for 10 times. Scale bar: 20 μm.

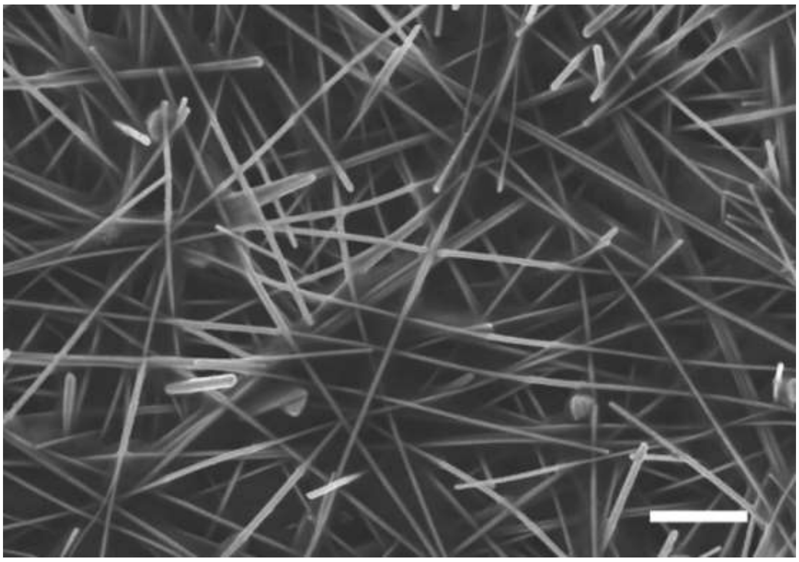

Figure S13. SEM diagram of the BAP electrode surface. Scale bar: 2 μm.

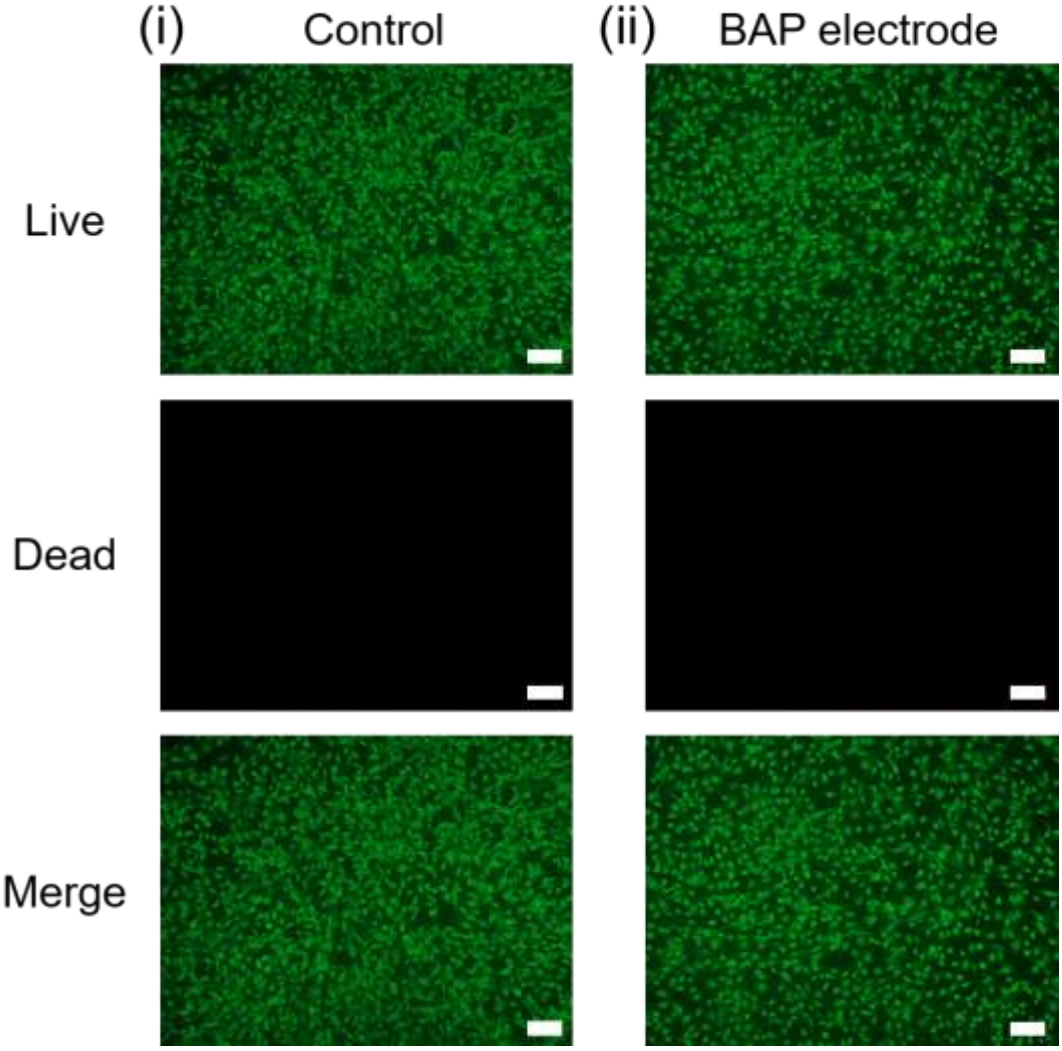

Figure S14. The live/dead fluorescence staining images of NIH3T3 cells (i) without and (ii) with BAP electrodes after 24 h of culturing.

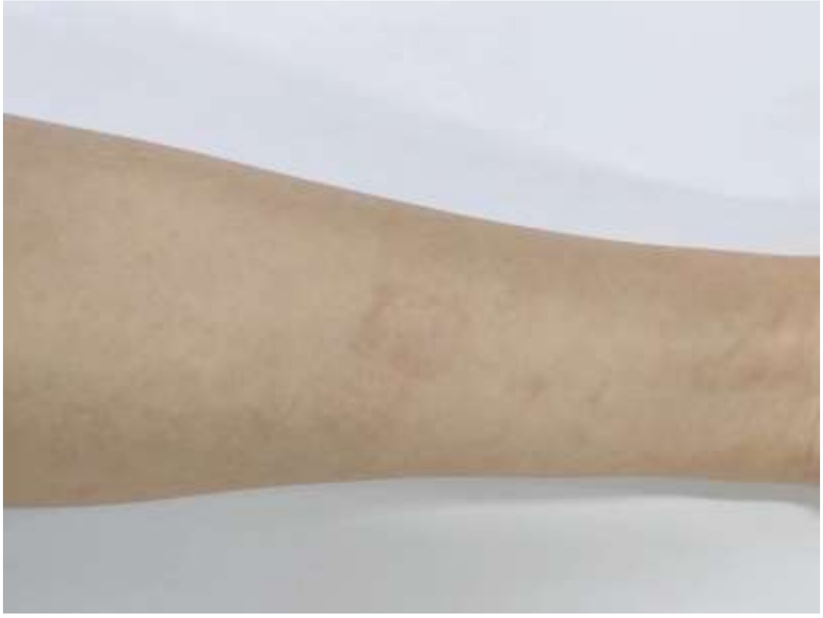

Figure S15. Optical photographs of skin when the BAP electrode was detached after being attached for 7 days.

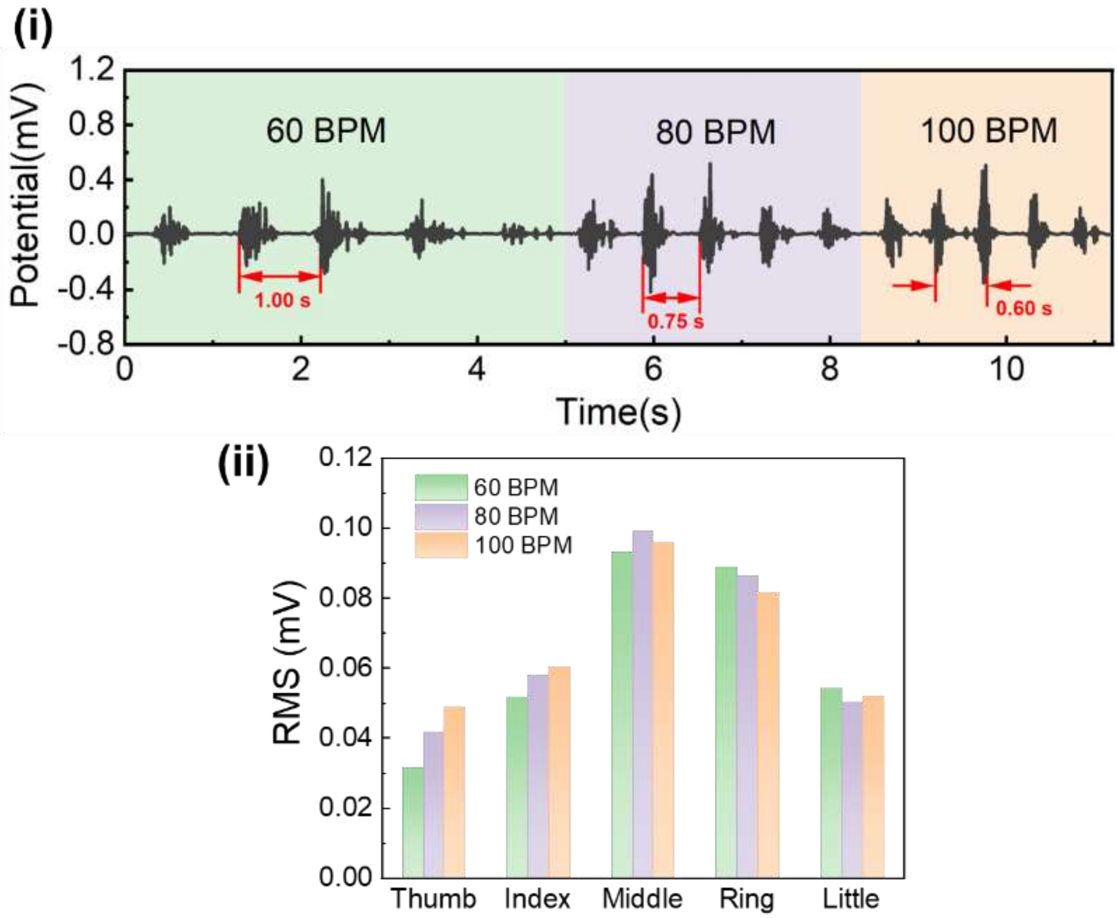

Figure S16. (i) sEMG signals collected when playing with different rhythms and its (ii) RMS value.
